# Supplementary material for: Video Laryngoscopic Intubation Using the King VisionTM Laryngoscope in a Simulated Cervical Spine Trauma: A Comparison Between Non-Channeled and Channeled Disposable Blades
Source: Diagnostics (Basel). 2020 Mar 3;10(3):139. doi: 10.3390/diagnostics10030139 (PMC7151076; doi:10.3390/diagnostics10030139)
Supplement: Supplementary file 1 [file diagnostics-10-00139-s001.pdf]

Cormack-Lehane and Percentage of Glottis Opening (POGO) scores.

Cormack-Lehane Score	Description	POGO Scores (%)
1	Full view of the cords	100
2	Most of the larynx visible	50–75
	Only posterior part of the larynx visible	25–50
3	Only epiglottis visible	0
4	Epiglottis and glottis not visible	0
